# Blood transcriptomics mirror regulatory mechanisms during hibernation—a comparative analysis of the Djungarian hamster with other mammalian species

**DOI:** 10.1007/s00424-023-02842-8

**Published:** 2023-08-05

**Authors:** Valeria Rojas Cuyutupa, Dominique Moser, Victoria Diedrich, Yiming Cheng, Jean-Noël Billaud, Elena Haugg, Dominique Singer, Jürgen Bereiter-Hahn, Annika Herwig, Alexander Choukér

**Affiliations:** 1 Laboratory of Translational Research ‘Stress and Immunity’, Department of Anesthesiology, LMU Hospital, Ludwig-Maximilians-Universität in Munich, Marchioninistr. 15, Munich, 81377 Germany; 2 Institute of Neurobiology, Ulm University, Albert-Einstein-Allee 11, 89081 Ulm, Germany; 3 Gene Center and Department of Biochemistry, Ludwig-Maximilians-Universität in Munich, Munich, Germany; 4 Institute for Diabetes and Obesity, Helmholtz, Munich, Neuherberg Germany; 5 QIAGEN Digital Insights, Redwood City, CA USA; 6 Division of Neonatology and Pediatric Critical Care Medicine, University Medical Center Eppendorf, Hamburg, Germany; 7 Institute for Cell Biology and Neurosciences, Goethe University Frankfurt, Frankfurt am Main, Germany

**Keywords:** Hibernation, Torpor, Transcriptomics, Ingenuity pathway analysis

## Abstract

Hibernation enables many species of the mammalian kingdom to overcome periods of harsh environmental conditions. During this physically inactive state metabolic rate and body temperature are drastically downregulated, thereby reducing energy requirements (torpor) also over shorter time periods. Since blood cells reflect the organism´s current condition, it was suggested that transcriptomic alterations in blood cells mirror the torpor-associated physiological state. Transcriptomics on blood cells of torpid and non-torpid Djungarian hamsters and QIAGEN Ingenuity Pathway Analysis (IPA) revealed key target molecules (TM_IPA_), which were subjected to a comparative literature analysis on transcriptomic alterations during torpor/hibernation in other mammals. Gene expression similarities were identified in 148 TM_IPA_ during torpor nadir among various organs and phylogenetically different mammalian species. Based on TM_IPA_, IPA network analyses corresponded with described inhibitions of basic cellular mechanisms and immune system-associated processes in torpid mammals. Moreover, protection against damage to the heart, kidney, and liver was deduced from this gene expression pattern in blood cells. This study shows that blood cell transcriptomics can reflect the general physiological state during torpor nadir. Furthermore, the understanding of molecular processes for torpor initiation and organ preservation may have beneficial implications for humans in extremely challenging environments, such as in medical intensive care units and in space.

**Supplementary Information:**

The online version contains supplementary material available at 10.1007/s00424-023-02842-8.

## Introduction

Torpor is one of the greatest mysteries in nature. To achieve an energy-saving survival mode during seasonal harsh environmental conditions such as extreme cold and food scarcity, metabolism is endogenously downregulated. Mammalian species which are capable to perform torpor reduce their metabolic rate (MR) and body temperature (T_b_) as well as heart and breathing rate [7, 35]. Aside from evading extreme winter-like conditions, some species inhabiting tropical areas in the southern hemisphere also perform torpor to overcome extreme drought, which is then termed estivation [49].

A period of daily torpor takes 3 to 12 h; however, when a consecutive torpor state lasts for more than one week it is called hibernation [26, 55]. A general differential characteristic within animals performing daily torpor or hibernation is body mass, whereby species exhibiting daily torpor tend to be small like rodents and hibernating species are large, such as bears [26]. Another characteristic is the achieved minimal T_b_, which is much higher in daily torpor species than in hibernators [35].

A small rodent that performs daily torpor is the Djungarian hamster (*Phodopus sungorus*). This dwarf hamster originally resides in eastern Kazakstan, southwest Siberia and in the Baraba steppe, where it is dependent on the ability to exhibit torpor to survive extreme winters [60]. The Djungarian hamster became a valuable animal model for torpor research because of its small size, relatively simple breeding as well as easy induction of seasonal winter acclimation by artificially changing the light-dark cycle at a moderate ambient temperature. Cold exposure or food restriction is not necessarily required for ultimate torpor induction [18, 36]. Thereby body weight reduces by 20 to 30% and its fur becomes white and well-insulating. In comparison to normothermic resting animals, MR is reduced to 44% and T_b_ can drop to a minimum of 12.5 °C for an average of 6 h per day [12].

The main regulators for torpor initiation and execution are yet still largely unknown. To investigate distinct changes at the genetic level, several studies focused on elucidating transcriptomic changes in organs that are suspected to be strongly involved in metabolic depression, such as the brain, liver, spleen, kidney, heart, and muscle in the torpid Djungarian hamster and other hibernating mammals [31, 34].

With regards to the understanding of torpor, blood was less systematically investigated thus far. Blood is a liquid organ that consists in general of erythrocytes, leucocytes, platelets, and blood plasma [4], with each compartment having its distinct functions such as gas transport, nutrient supply, immune defence, coagulation, thereby maintaining body homeostasis [70]. The amount of blood volume constitutes 3–8% of each species´ body volume [38] and its ever-moving circulating system through all vessels and organs ensures nutritional supply and signaling to the respective target organs. On the other side, the release of organ-specific markers into the blood allows insight into their functional state and health condition [53].

Hence, blood is considered not only a vehicle, which transports blood cells and humoral components through different organs but in turn represents a dynamic, species-independent reliable source for the determination of current physiologic states. Based on this, the hypothesis was raised that transcriptomic alterations in blood cells may reflect torpor-associated transcriptomic changes in the whole body. In the present study, transcriptomic changes in the blood cells of Djungarian hamsters at the nadir of spontaneous daily torpor were compared with literature-derived data on the transcriptome of solid organs and tissues from different torpid and hibernating species.

The identification of a distinct set of genes that are involved in torpor initiation or execution may not only help to decipher the physiological processes involved in, but it may add to the development of strategies to induce torpor in non-hibernators such as humans, which may have diverse applications e.g., in intensive care medicine and during space travel.

## Materials and methods

### Experimental animals

#### Breeding and housing

Nine Djungarian hamsters (*Phodopus sungorus*) were bred and raised according to an outbred crossing scheme in the indoor breeding colony at the Institute of Neurobiology (Ulm University, Germany) in accordance with the local ethics committee (z.231-1, RP Tübingen). The ambient temperature was maintained at 20 ± 1 °C. Artificial light (200-250 lux) was provided 16 h per day in summer-like long photoperiod and 8 h per day in winter-like short photoperiod. A permanent red-light LED (<5 lux) permitted sampling during the dark phase. Tap water and food (Altromin hamster breeding diet 7014, Lage, Germany) were provided *ad libitum*, supplemented by cucumber, oat flakes, and sunflower seeds once a week. Adult hamsters were single housed in Makrolon Type III cages (820 cm^2^) with wooden bedding and tissue as nesting material.

Besides the time of day, T_b_ was used to identify the correct sampling timepoint. To measure body temperature and locomotor activity, a radiofrequency transmitter (Data Sciences International (DSI), Harvard Bioscience Inc., St. Paul, MN, United States) was implanted intraperitoneally under isoflurane anaesthesia (2.5% and 1 ml/min for induction, 0.75–2.0% and 0.4 ml/min for maintenance) and carprofen analgesia (5 mg/kg, i.p.; Rimadyl, Zoetis Deutschland GmbH, Berlin, Germany). Recovery from surgery was supported by additional oat flakes, sunflower seeds, cucumber, and nesting material. Body mass, coat care, posture, and behaviour were monitored daily for about 7 days. Experimental and surgical procedures were approved by the Regional Council of Tuebingen, Germany (1411).

#### Key data of hamsters

All hamsters (n=9) were acclimated to winter-like short photoperiod (SP) with 8 h of light per day from the age of 16 ± 3 weeks, were implanted with DSI transmitters at the age of 30 ± 3 weeks, and sacrificed at the age of 32 ± 4 weeks. During the 16 ± 2 weeks of SP acclimation, they reduced their body mass by 24 ± 8% from 37 ± 5 g to 28 ± 4 g.

#### Sampling scheme

All nine SP-acclimated hamsters were observed to express spontaneous daily torpor, which is a drop of T_b_ <32 °C for at least 30 min. On the day of sampling, five hamsters expressed torpor (hypothermic animals (HT)) and the other four hamsters did not and served as normothermic controls (NT). Blood sampling was performed in both groups at *zeitgeber* time point 4 (ZT4), during torpor nadir, whereby ZT00 referred to the beginning of the light phase. For sampling, the hamsters were sacrificed by carbon dioxide inhalation within their home cage. Via puncture of the right heart ventricle, 0.5 ml of blood was collected in EDTA-containing microvettes (Sarstedt, Nümbrecht, Germany), placed immediately on ice and centrifuged at 4 °C for 10 min at 2,000 rpm. Blood plasma was removed, and blood pellets were stored at −80 °C until RNA purification. Analyses of core body temperature, locomotor activity, body mass, small intestinal tissue, and fur index in a subset of hamsters have already been published [33, 52].

### Blood sample processing and Next Generation Sequencing

#### RNA isolation and quantification

Frozen blood pellets were thawed in 400 μl lysis buffer and RNA was isolated using the Mini NucleoSpin RNA Blood Kit according to the manufacturer’s instruction (MACHEREY-NAGEL, Düren, Germany). RNA concentrations were quantified by NanoDrop fluorometer (Thermo Fisher Scientific, Waltham, MA, USA), and RNA integrity was assessed by Qubit and RNA IQ Assay (Thermo Fisher Scientific, Waltham, MA, USA). Samples passed internal quality control with a purity of 2.08–2.11 and the RNA integrity number was 8.85 ± 0.76.

#### Next generation sequencing

Additional quality controls using fragment analyser, random primed cDNA synthesis, and RNA sequencing by Next Generation Sequencing (NGS) were performed at Eurofins Genomics Europe Sequencing GmbH (Konstanz, Germany). NGS was done on an Illumina HiSeq 2500 platform in a paired-end configuration of 2 x 150bp with a minimum of 30M reads per sample.

### Bioinformatics

#### De novo assembly of *P. sungorus* transcriptome

Each fastq file underwent quality control using fastqc 0.11.9 (http://www.bioinformatics.babraham.ac.uk/projects/fastqc). The adapters and low-quality bases were removed with trimmomatic 0.39. Since no annotated genome of Djungarian hamster was available, it was necessary to perform a *de novo* assembly of the transcriptome. The best practices for *de novo* transcriptome assembly with trinity 2.8.6 was followed (https://informatics.fas.harvard.edu/best-practices-for-de-novo-transcriptome-assembly-with-trinity.html) to generate the reference contigs of Djungarian hamster transcriptome using all the sample fastq files merged together. BUSCO v5 was used to assess the annotation completeness using the “-m transcriptome –auto-lineage-euk,” which resulted in 99.3% completion. In order to assign the ortholog, each contig was translated into 6 possible peptides and blasted against the human proteome. The human gene symbols (HGNC) were assigned for each contig based on the best match of the blast bitscore cutoff 50. To obtain the gene expression quantification, the samples were aligned to the assembled transcriptome using histat2 and quantified with custom PERL scripts.

#### Differential gene expression analysis and Ingenuity pathway analysis

Differential gene expression of HT versus NT was assessed using DESeq2. Differentially expressed genes were imported to IPA (https://digitalinsights.qiagen.com/products-overview/discovery-insights-portfolio/analysis-and-visualization/qiagen-ipa/) [42], for biological analysis using the cutoffs: *p* value ≤0.05 and fold change (fc) ≥|1.25|. Using the IPA Regulator Effects feature, target molecules can be chosen which are the most relevantly regulated genes in the dataset. The algorithm used by IPA for the regulator effects tool connects Upstream Regulators, Downstream Effects, and the molecules in the dataset to generate a hypothesis that can explain how the activation or inhibition of a regulator affects the expression of the target molecule and the impact of molecular expression on metabolic functions and diseases. For this process, one or more iterations are used to merge the results of these regulators. Networks are merged only if the overlap of targets has a significant statistical value (Fisher’s exact test *p* value of <0.05). Moreover, at the same time, possible mechanisms behind a phenotype can be identified and the biological impact of the regulated molecules can be determined.

### Literature search protocol

**Fig. 1:**
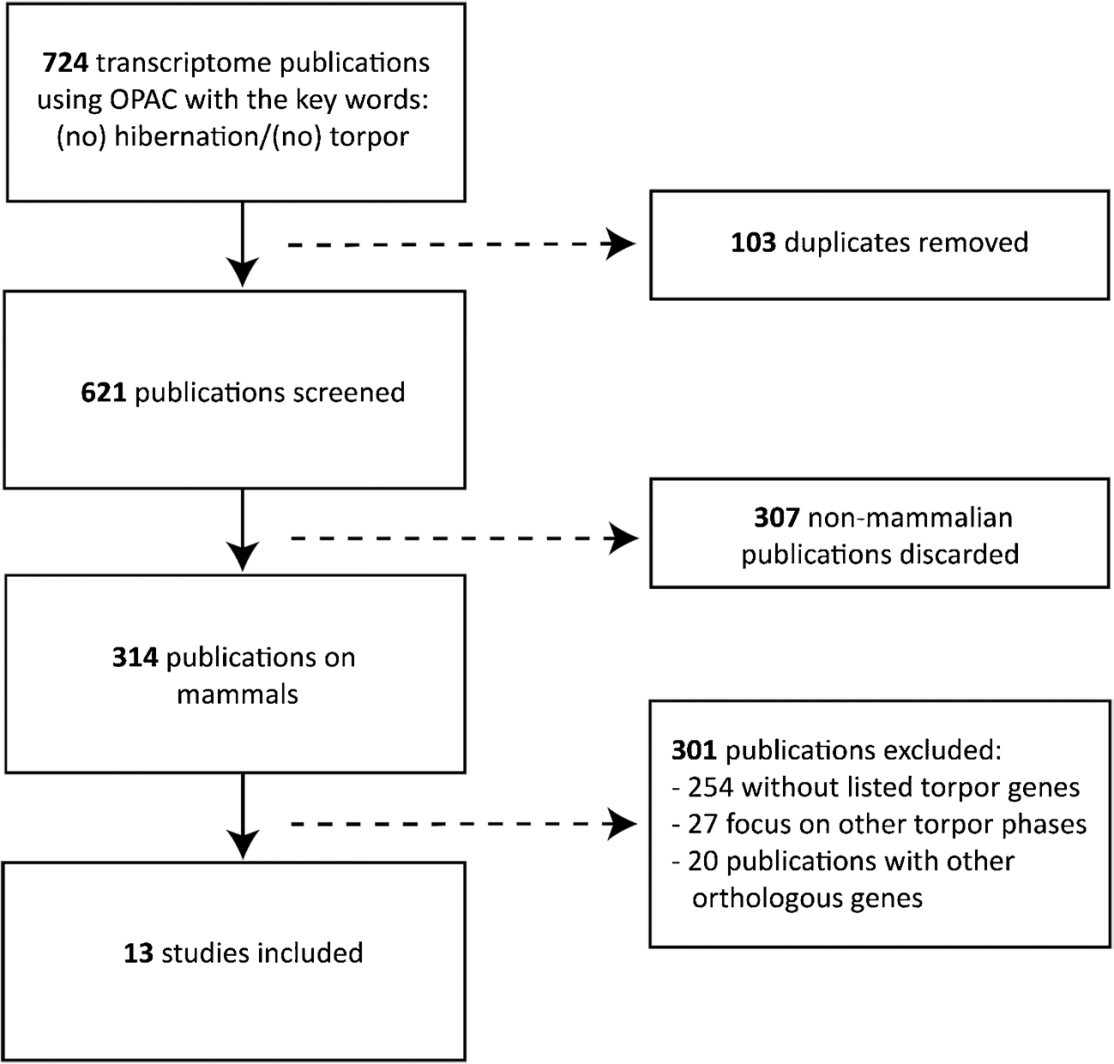
Study flow diagram outlining literature selection for comparative literature analysis. Based on 724 publications identified in the OPAC database, 314 publications on mammals were reviewed in total. For the final analysis, detailed descriptions were only available in 13 studies that were hence evaluated, and the described genes were compared to TM_IPA_

For collecting existing data on transcriptomic changes during torpor, irrespective of order, species, and organ, an extensive literature review was performed using the keywords “Transcriptome,” “Torpor,” and “Hibernation” in the meta-databank Online Public Access Catalogue (OPAC) of the Ludwig-Maximilians-Universität (LMU) in Munich. For this analysis, 314 papers were screened and only considered when a comparison was drawn between “Torpor/Hibernation” or “no torpor/no hibernation”, respectively. Altogether, data from 13 publications were extracted for the comparison of transcriptional changes (Fig. 1).

### Gene clustering

The classification of genes was performed according to the biological process with the Gene Ontology program of Panther (Protein ANalysis THrough Evolutionary Relationships, http://www.pantherdb.org, version 16.0, California). Panther is part of the Gene Ontology Phylogenetic Annotation Project.

### Graphic edition

All images were designed with the graphic editor Adobe Illustrator (Adobe Inc., 2018).

## Results and discussion

Blood was sampled from Djungarian hamsters at torpor nadir and from time-matched controls on a torpor-free day.

Differential gene expression analysis retrieved a considerable number of up- or downregulated genes (11,422) out of which target molecules were identified according to the cut-offs (TM_IPA_). The subsequent comparative literature analysis was based on these TM_IPA_. To identify relevant experiments from the literature, 314 publications were screened that dealt with transcriptomic changes in torpor considering only those hits including the keywords “torpor/hibernation” or “no torpor/no hibernation” respectively in mammals. Among these publications, those were considered for comparative literature analysis which provided a detailed list of regulated genes and which corresponded to TM_IPA_ (215 genes in total). Reports dealing with other (orthologous) genes, which were not found within TM_IPA_, were excluded (Fig. 1). Based on these criteria, 13 studies included detailed information and were hence subject in the analysis (Suppl. Table 1). TM_IPA_ and described genes in literature were compared regarding their direction of regulation (up- or downregulation). Among the 215 identified genes, 148 (68.8 %) genes showed matches in torpor regulation.

### Gene expression similarities across different species and organs

**Fig. 2:**
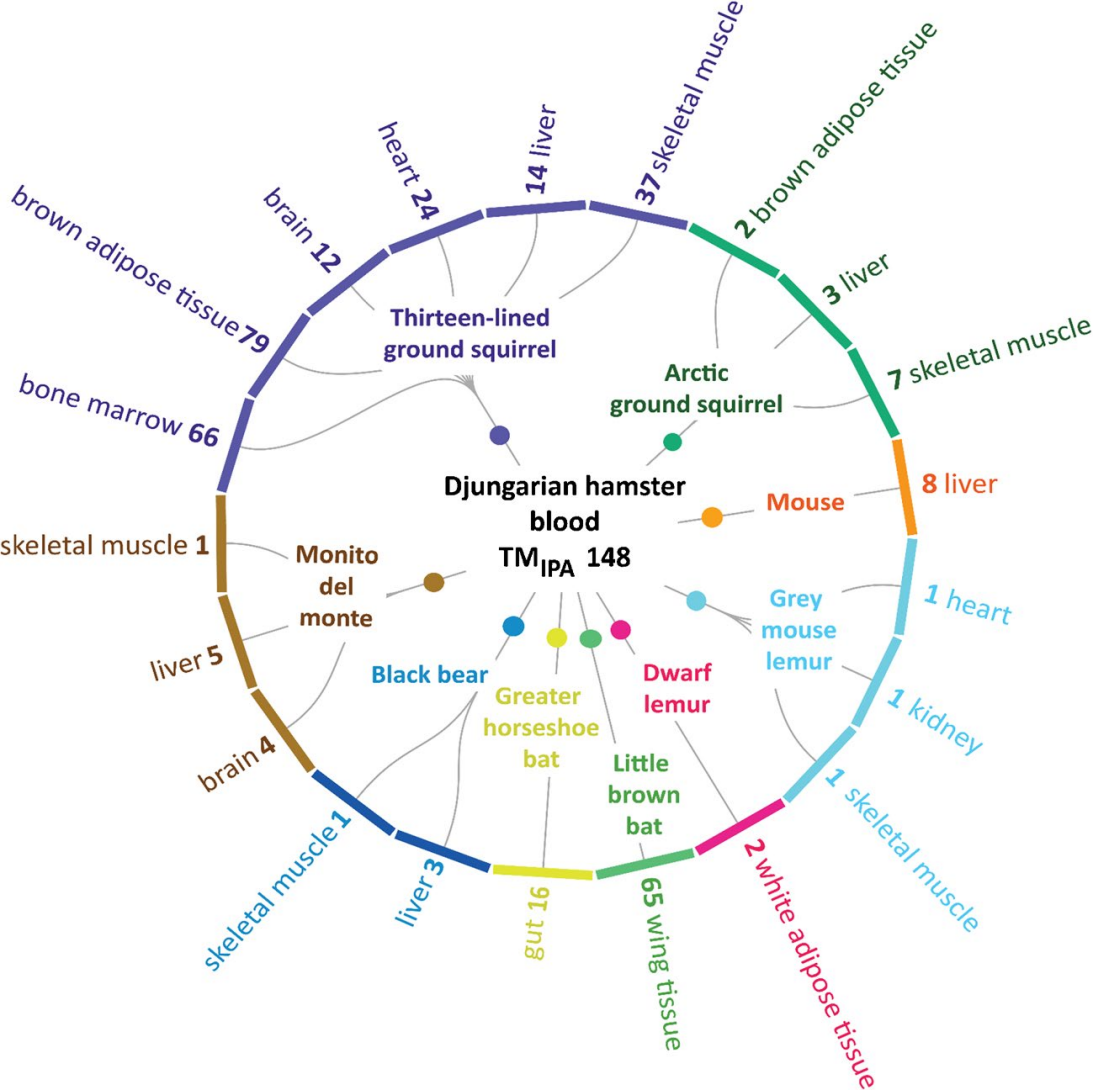
Radial diagram showing the distribution of 148 matches of target molecules (TM_IPA_) and data from literature research. TM_IPA_ from the transcriptome of Djungarian hamsters in ZT04 were identified by IPA and compared to transcriptomic data from hibernating mammals described in the literature. The digits next to the organs represent the number of genes found in the respective organ. The same gene can be affected in different organs of the same species

**Fig. 3:**
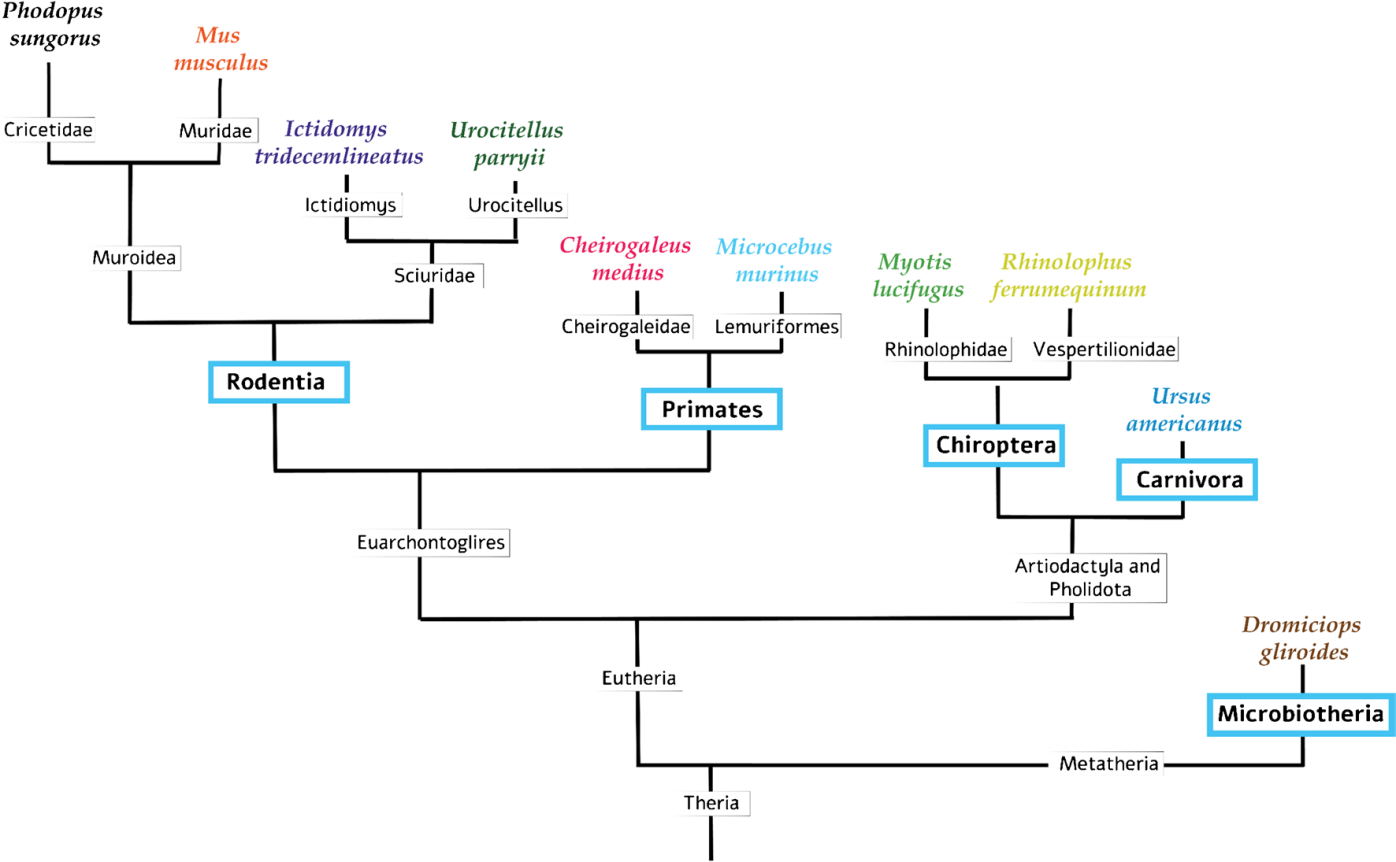
Simplified phylogenetic tree demonstrating the distance in phylogenetic relationship of the orders (blue boxes) the analyzed species belong to

The torpor-regulated matching genes that have been reported in the literature derived from research on nine different mammalian species from the orders Rodentia, Primates, Chiroptera, Carnivora, and Microbiotheria and in total ten different organs (Figs. 2 and 3). Most matches with Djungarian hamsters were found for the thirteen-lined ground squirrels (*Ictidomys tridecemlineatus*), for which similarities were described for 122 genes in altogether six different organs. The second most matching genes were identified for the little brown bat (*Myotis lucifugus*) with 65 genes in one organ. This was followed by the greater horseshoe bat (*Rhinolophus ferrumequinum*) with 16 genes in one organ, the arctic ground squirrel (*Urocitellus parryii*) with eight genes in three organs, the house mouse (*Mus musculus*) with eight genes in one organ and monito del monte (*Dromiciops gliroides*) with eight genes in three different organs, the black bear (*Ursus americanus*) with four genes in two different organs, the dwarf lemur (*Cheirogaleus medius*) with two genes in one organ and finally the grey mouse lemur (*Microcebus murinus*) with one gene in three different organs, respectively (Fig. 2). Regulated genes from each species and organ that matched with TM_IPA_ derived from torpid Djungarian hamsters are displayed in Suppl. Table 2.

### Phylogenetic relationship between the orders

Remarkably, the order classification of these species showed very different degrees of phylogenetic relationship (Fig. 3) and torpor modes.

In the order Rodentia, corresponding genes were found in three species. Among them were the thirteen-lined ground squirrel (*Ictidomys tridecemlineatus*) and the arctic ground squirrel (*Urocitellus parryii*). Both species hibernate for a period of six to nine months where their drastic metabolic reduction results in a T_b_ decrease down to values ranging from 2 to 10 °C. In this state, their heart rate drops to 3–10 beats per minute (bpm) (in active state 200–300 bpm), oxygen consumption reduces to 2–3 % of normal levels and MR is reduced by 98 % [59]. During the hibernation period, thirteen-lined ground squirrels arouse every ten days to eat stored food and urinate, whereby their T_b_ rises from 8 to 37 °C in less than 3 h [11]. Arctic ground squirrels (*Urocitellus parryii*), however, rewarm in regular episodes of arousal spontaneously to euthermic levels (36–37 °C) and maintain that temperature for 15–24 h before re-entering torpor [69].

The only non-hibernator by nature in this phylogenetic order is the house mouse (*Mus musculus*). A torpid state can be induced in house mice by a negative energy balance through food restriction and a moderate decrease in ambient temperature below the thermoneutral zone. In this so-called synthetic torpor state [62], the mice conserve energy by reducing MR by 45.1 ± 4.6 %, immobility, decrease in sensory perception, respiratory rate, and heart rate [37, 62].

Within the primate order, similar gene expression regulation during torpor/hibernation was found in the fat-tailed dwarf lemur (*Cheirogaleus medius*) and the grey mouse lemur (*Microcebus murinus*). In the highly dry seasonal climate of Madagascar, fat-tailed dwarf lemurs “hibernate” for up to seven months in order to reduce energy in times of extreme drought and food shortage. Over many weeks or even months, their T_b_ is close to ambient temperature, causing daily fluctuations up to 20 °C (between 10 and 30 °C) [14]. Dwarf lemurs can easily double their body weight before hibernation through increasing white adipose tissue (WAT), which then serves as an energy reservoir during prolonged period of physical inertia [13]. In contrast, grey mouse lemurs (*Microcebus murinus*) exhibit daily torpor. They live in the dry deciduous forests of Madagascar and are able to enter torpor when dryness sets in and food and water are scarce [57]. These animals reduce their T_b_ in a range of 7.8–29.1 °C. This large variability in T_b_ during torpor is due to strong variations in ambient temperatures ranging from 7.2 to 18.5 °C [58]. It is interesting to note here, that lemurs are the species closest related to humans that undergo natural hypometabolism [3, 19].

In the order Chiroptera, similarities in transcriptomic changes during torpor were found in the little brown bat (*Myotis lucifugus*), a small insectivore that lives throughout North America, from Alaska to Mexico [21]. The northern populations hibernate from September to May and the southern populations from November to March. The little brown bat does not migrate during the change of seasons. The duration of the hibernation season of these bats depends on latitude and takes place for five to eight months per year in caves and mines that are cold (−4 -to 13 °C), humid (>90% relative humidity), and draughty [51].

The other bat species is the greater horseshoe bat (*Rhinolophus ferrumequinum*), which has a wide distribution across Europe, Africa, and Asia and has become a model organism for studying bat hibernation. Within this species, hibernation takes place from October to April with irregular inter-bout arousals. The bats hibernate in cold underground sites during winter [16] for up to eight months without a food supply and their T_b_ can be reduced to 10 °C and lower [21].

Within the order of Carnivores, transcriptomic similarities with Djungarian hamsters were identified in the black bear (*Ursus americanus*) which hibernates for three to six months. However, in this special hibernation state the animals are still able to move, and they remain conscious, however without defecating, eating, or urinating. Although black bears do not reduce their T_b_ as drastically as rodents (from 36 to 30 °C), their MR is reduced by 20–50% and after hibernation, it takes two to three weeks for them to recover to their normal T_b_ [63].

The phylogenetically most remote order, in which transcriptomic similarities were found is the order of Microbiotheria. Here monito del monte (*Dromiciops gliroides*), a South American marsupial, exhibits short periods of daily torpor in summer and slightly longer periods of torpor or hibernation in winter. This allows these small mammals to save up to 60 % of their daily energy needs in the cold season and to reduce MR by around 90% [47].

Altogether, a considerable number of transcriptomic alterations are similar in the Djungarian hamster during daily torpor nadir and other mammals in different states of metabolic downregulation, irrespective of organ type, species, order, and the mode of torpor/hibernation (Fig. 2 and Fig. 3).

However, this comparative approach is based on a collection of data derived from a literature search with defined keywords. It cannot be excluded that published data on other hibernating species were not retrievable as by the keywords applied, or have not been published by the date of search.

### Clustering the matching genes into physiological categories

**Fig. 4:**
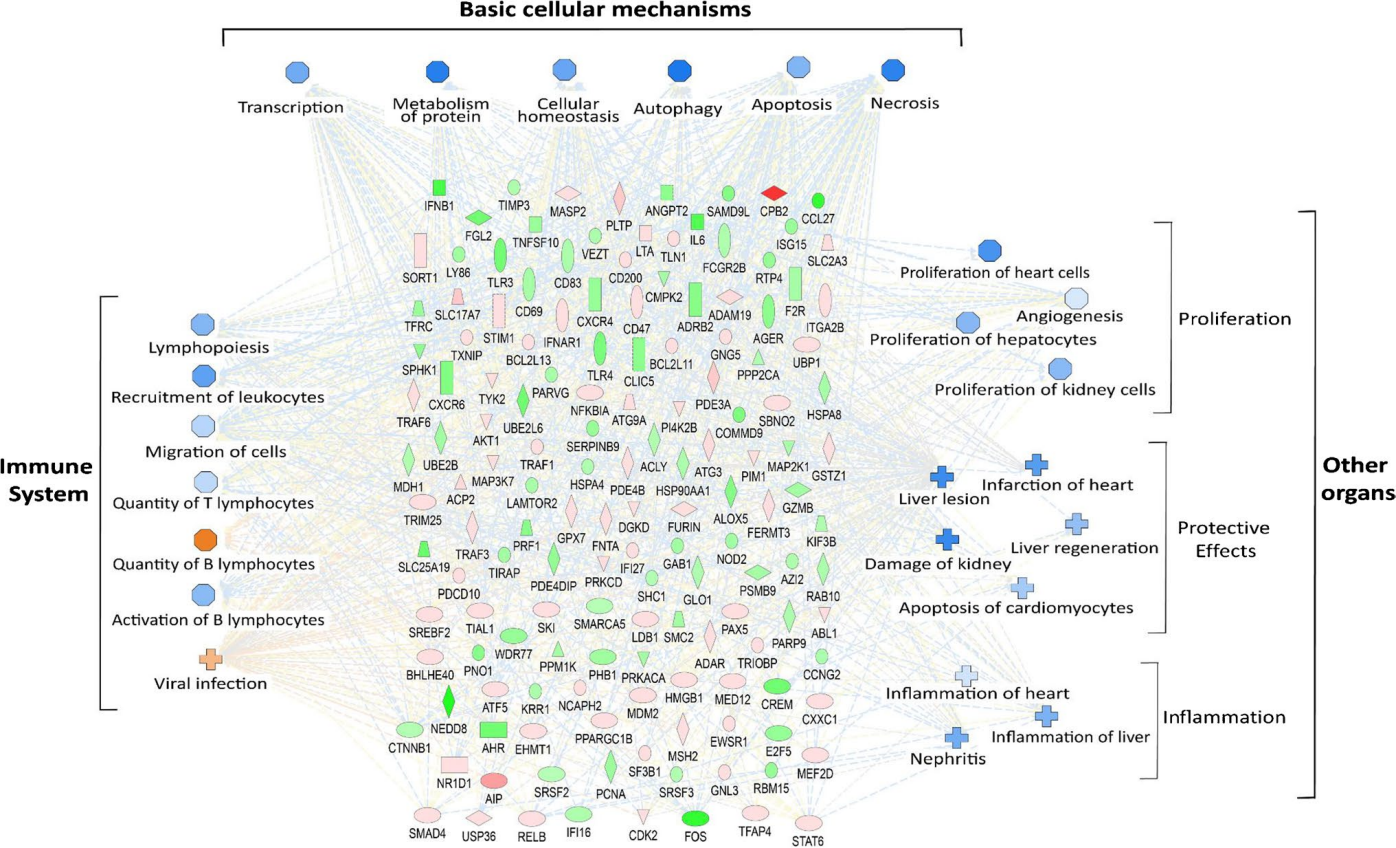
Predicted network downstream effects based on upstream regulating functions of 148 matching TM_IPA_. Regulated genes (green: decreased measurement, red: increased measurement) were processed by the *network analysis* tool of IPA and the predicted downstream effect was assessed with regard to *basic cellular mechanisms*, *immune system*, and *other organs*. Blue symbols describe predicted inhibition and orange symbols describe predicted activation. Intensity of the respective colour mirrors the magnitude of gene expression regulation or downstream prediction, respectively. Gene symbols are depicted according to IPA and are categorized according to their functional characteristics, e.g., kinases or transcriptional regulators. Octagons represent function and crosses represent diseases

To obtain insights into the involvement of the matching key target molecules (TM_IPA_) in homeostasis and functional properties, genes were clustered by means of the IPAs network analysis tool into distinct physiological categories, which are known to also have relevance in torpor. By this, the potential outcomes of the up- or downregulated TM_IPA_ were predicted in relation to cellular processes and organ (dys-)functions. The first category was termed *basic cellular mechanisms*. It includes basic physiologic processes, which are crucial for cellular homeostasis and cell survival. The second category comprised grossly the *immune system* with a focus on both its maintenance and distinct functions. The third category encompassed the immediate protective effect of TM_IPA_ regulation on *other organs*, with the heart, kidney, and liver being highly affected representatives (Fig. 4).

During torpor and hibernation, *basic cellular mechanisms* are drastically shut down to save energy and assure survival. Network analysis of the 148 TM_IPA_ predicted a general inhibition of *transcription* and *protein metabolism*. This corresponds with reports describing the need for a balanced transcription depending on the demands of gene products, since protein biosynthesis is an energetically expensive cellular process [17, 64]. Moreover, the shut-down of the transcription machinery is believed to be associated with low temperatures, because cold depresses moderate transcription initiation and more severely elongation, making transcriptional depression during torpor a reversible process [64]. However, the Djungarian hamsters in this study were kept at constant ambient temperatures of 20 ± 1 °C, suggesting energy saving to be the main reason for downregulated transcription. The involvement of TM_IPA_ in diverse cellular processes also resulted in a predicted inhibition of *cellular homeostasis* and *autophagy* during torpor. For a physiological equilibrium within cells, the function of signalling pathways, timely control of ion pumps, and proper protein turnover are decisive [46, 54, 61]. Modulations of central regulators such as mTOR and AMPK are known mechanisms that control cell growth and survival during torpor [44, 61]. *Autophagy*, in which cells degrade their own proteins and organelles in order to get rid of dysfunctional proteins and to provide metabolites was in contrast to IPA prediction, reported to be activated during torpor [17]. However, most analyses on autophagy were performed at the early phase of the arousal process, thereby mirroring rather the high energy demands during arousal and activation of a protein quality control system to clear damaged proteins than the situation in torpor nadir [17, 65]. The last two processes in this first category, *apoptosis,* and *necrosis***,** were in line with reports of limited apoptosis and fostered pro-survival processes by the induction of anti-apoptotic mechanisms, altered signal transduction, reversible post-translational modifications of proteins, specific microRNAs, and anti-oxidative protection [1, 56]. The same mechanisms also add to the prevention of necrotic tissue damage [7]. Altogether, the predictions of IPA regarding inhibition of *basic cellular mechanisms* in torpor based on TM_IPA_ demonstrate that common changes, which are valid for the whole organism can be displayed by the blood cell transcriptome.

Since blood cells were the samples for the present transcriptome analyses in Djungarian hamsters, the second category dealt with TM_IPA_-derived predictions on inhibition or activation of *immune system*-associated processes (Fig. 4). It is broadly acknowledged that torpor induces a state of immune dormancy in which innate and adaptive immune cell functions are decreased in order to conserve energy [5, 7, 39]. Immune processes, which IPA predicted to be inhibited were well in accordance with other publications. According to the expression profiles of TM_IPA_, overall *lymphopoiesis* was inhibited, which was also reported by Bouma and colleagues [5]. The retainment of approximately 90% of the immune cell repertoire during torpor in the gut and spleen (predominantly lymphocytes) as well as in the lung (predominantly granulocytes) [5, 39] was well reflected by IPA, since it predicted an inhibited *recruitment* and *migratory* potential of leukocytes. Again, low T_b_ is regarded as the main driver of regulated immune cell abundance and function during torpor/hibernation [6]. However, predicted low *T lymphocyte quantity* in the Djungarian hamster setting indicates at least to a certain degree temperature-independent mechanisms. Interestingly, the *quantity of B lymphocytes* was predicted to be increased despite lymphocyte retention, which corresponds with findings of an increased proportion of B lymphocytes in the intestinal lamina propria of hibernating bats and ground squirrels [5]. This does not only confirm IPA predictions, it shows that transcriptomic alterations in blood cells may also reflect the situation in the gut during torpor. The limitation in immune cell availability during torpor may result in an increased susceptibility towards infection with pathogens such as fungi and viruses [5]. Furthermore, *activation of T lymphocytes* was predicted to be decreased during torpor. This can be verified by observations made by Novoselova et al., where T lymphocytes derived from hibernating ground squirrels displayed a reduced proliferative capacity upon Concanavalin A stimulation [48]. Moreover, the downregulation of several genes involved in antiviral immunity led to a prediction of higher rates of *viral infection* during torpor. This is mirrored by reports of reduced stimulation-induced IFNγ production and depletion of activated CD8^+^ T cells in hibernators [5, 41]. Interestingly hibernating mammals do not suffer from viral infection or re-activation, which is most likely attributed to a virus dormancy that comes along with the reduced metabolic activity of the host [27, 28].

Of note, genetic alterations that led to changes in the immune cell level were documented for all organs within the reviewed literature. It cannot be excluded, that immune cells were present in the analysed tissues, which may have added to the high concordance. However, since in the majority of studies (7 of 13) organs were flushed before preparation, the amounts of immune cells are expected to be low. These data show that a distinct set of genes is affected in blood cells and throughout several organs that reflect impaired immune cell capacities during torpor.

In torpor or hibernation, *organs* are capable to resist tissue damage despite unavoidable cell stress. Thus, the third category describes the IPA-predicted effects of gene regulation identified in blood cells on the *heart*, *kidney*, and *liver* (Fig. 4).

Altogether, *proliferation* was predicted to be inhibited. Moreover, the transcriptional profile indicated reduced angiogenesis. This corresponds well with the observation that cell proliferation as a very energy-consuming process is shut down during torpor/hibernation and mirrors functional adaptations during torpor/hibernation. A high proportion of cells reversibly exits the cell cycle during hibernation and remains arrested in the G_0_-phase until arousal [17]. In the case of the heart, cardiomyocytes become hypertrophic in G_0_ to manage the increased workload during torpor due to an increased viscosity of the blood, which is accompanied by reduced vascular elasticity [68]. Kidney function is strongly downregulated in torpor with almost no glomerular filtration or urine output. This is mainly due to hypothermia-mediated renal vasoconstriction and reduced renal blood flow because of infrequent cardiac output. However, no structural changes occur, neither growth-promoting nor degrading processes, which ensures a fully maintained function directly after arousal [40]. Reduced proliferation of hepatocytes within the liver is a result of a reversible cellular quiescence of these cells during torpor [67]. In active (summer) conditions, hepatocytes display a high proliferative capacity to regenerate quickly in the case of tissue damage.

*Inflammatory processes* in the heart, kidney, and liver were also predicted to occur more rarely (Fig. 4). Due to the depressed functional capacities of both the particular organ and the immune system, lower levels of inflammation were expected. The reduced expression of TLR4, which was found among TM_IPA_ and which is highly abundant in heart inflammation [20] suggests a reduced inflammatory potential in heart tissue during torpor. Moreover, immune activation and inflammatory signalling pathways in the liver were shown to be strongly suppressed during torpor [43].

Altogether, local adaptations of organs towards the reduction of MR and T_b_ result in *protection* from organ damage. Animals capable to perform torpor developed mechanisms, which induce resistance against hypothermia-induced cell damage, ionic overload, and tissue injury due to an ischemic state and increased perfusion upon arousal, whereby the entire genetic program seems to be activated independently of ambient temperatures. In non-hibernators, an extensive drop in T_b_ would lead to cardiac arrhythmias, which can result in heart infarction, sudden cardiac death, or causes ischemic reperfusion (IR) injury upon arousal [68]. Adapted signalling pathways enable the regulation of ion transporters, which control cardiac contraction and relaxation, and the elevated production of anti-oxidative enzymes, which protect from cardiac injury during and after torpor/hibernation together with an upregulation of anti-apoptotic genes [30, 68]. Kidney adaptations likewise permit to withstand low T_b_ and organ perfusion as well as reperfusion by the increase of antioxidant enzymes and the downregulation of pro-apoptotic processes [40]. Hepatocytes and the whole liver as such are also protected against damage by torpor-associated conditions and IR, which is mainly attributed to the upregulation of antioxidants and anti-apoptotic proteins [43, 50].

Aside from this, a low occurrence of glucose metabolism disorders and brain lesions was predicted on the basis of TM_IPA_ (data not shown), for both of which hibernating animals were observed to be resistant during deep torpor [2, 15].

Altogether, these data suggest that the suppression of energy-consuming basic cellular processes, downregulation of pro-inflammatory immune responses, and organ protection by predominantly suspending pro-apoptotic processes and fostering production of antioxidants allow one to go into torpor/hibernation without any signs of tissue damage upon arousal. Moreover, these data further corroborate that transcriptomic alterations which are required for performing torpor/hibernation in general and on the specific organ level can be detected and mirrored by blood cells.

The assignment of each cellular process and organ (dys-)function to the respective set of genes is listed in Suppl. Table 3.

### Implications of transcriptome discoveries in blood during torpor

The findings resulting from the differential gene expression analysis, the subsequent comparative literature research, and the IPA network analysis demonstrate that common regulatory mechanisms at the genetic level exist that are independent of species, organ, and mode of metabolic reduction (torpor or hibernation). Moreover, the analysis of torpor-specific transcriptomic alterations in blood cells allows for analyzing changes in a standardized manner across different science groups and experimental questions. The analysis of solid organs imperatively requires to sacrifice of the animal and always bears the risk to induce changes due to operational procedures such as the way of organ removal and tissue processing.

Using blood as a sample specimen for torpor-associated transcriptomic changes within the whole body would allow monitoring transcriptional alterations in hibernation and daily torpor in particular which are known to be very dynamic processes. The inter-bout arousals which are observed among the majority of hibernating species are characterized by a rapid shift in metabolism and body temperature. To sustain these radical changes and to remain homeostasis, fast transcriptional adjustments by *de novo* transcription are required to meet the specific need in each respective phase of torpor/hibernation [25, 29].

### Translations from hibernation physiology to human health

Learning from the blood transcriptome of hibernating species can help to deepen the understanding of torpor which likely bears great potential for human medicine and spaceflight. For example, the discovery of brown adipose tissue (BAT) in humans and the understanding of BAT thermogenesis derived from studies on appropriate hibernating mammals may help to identify an endogenous therapeutic target to treat obesity and diabetes [2]. Hibernators are natural model organisms for physiological obesity without detrimental health consequences. Understanding the underlying mechanisms, which are supposed to occur at the proteomic rather than on the genetic level, may help to identify responsible biomarkers for human sedentary lifestyle-mediated diseases [24] and to develop counteracting therapeutic strategies. Torpid/hibernating rodents thereby evolved various strategies of biochemical changes. Thirteen-lined ground squirrels for instance display reversible insulin resistance when undergoing hyperphagia before hibernation season to increase their body fat storage [66]. Elucidating the mechanisms of reversed insulin resistance might add to curative approaches for diabetes mellitus type 2. In Djungarian hamsters, however, torpor bouts are initiated by lowering body mass, fat stores, and leptin levels of which understanding the underlying mechanisms may support the development of anti-obesity therapies [18, 22]. Because of their special hibernation mode and only moderate decrease in T_b_, bears may represent an appropriate translational model for humans. In preparation for hibernation, these animals exhibit extensive hyperphagia and gain around 30 % of body mass compared to spring without showing any signs of reduced insulin sensitivity or metabolic disorders. Moreover, the month-long immobility during hibernation, which would cause severe bed sores, loss in bone mineral density, and sarcopenia in humans, does not cause any detrimental effects on the musculoskeletal system in bears [23, 32, 45].

In addition to that, protective processes occurring in hibernators in general may have strong implications in human critical care medicine such as their resilience to kidney injury and hypoxia tolerance, which may be meaningful during organ transplantation and severe organ damage. Moreover, understanding metabolic reduction in a hypoxic microenvironment may add to new treatment options for cancer diseases [9].

For long-duration manned spaceflights, a targeted metabolic downregulation would have a game-changing character. Water and food intake could be reduced up to 75% and the payload of the spacecraft, in particular the oxygen supply, could be reduced. Detrimental effects on crew health could be alleviated by reducing astronauts´ mental stress caused by long-term isolation [10] and radiation susceptibility [8] as shown for hibernating species [62].

## Conclusion

In this study, differential gene expression analysis as derived from the blood of Djungarian hamsters during torpor nadir was subjected to a comparative literature analysis on transcriptomics in different organs of other mammals expressing daily torpor or hibernation. Based on the regulatory similarities identified, blood cells were demonstrated to reflect changes in the whole physiological state at the transcriptional level and thus represent a source for monitoring torpor. However, the present analysis had a pure descriptive character and did not distinguish between active regulatory effects and passive effects that occur as a consequence of the hypometabolic state. Moreover, such comparative studies on transcriptional alterations would benefit from analyses of blood cells and several organs derived from the same animal, however, this endeavour is beyond the scope of the present study and represents a worthwhile topic for future investigations.

Nevertheless, it is reasonable to state a principal independence of species, torpor mode, and the organ involved in the different adaptive processes. This would allow the investigation of torpor-like states or the targeted induction of metabolic reduction in humans, which might have great implications to mitigate extreme conditions in medicine and enable crewed missions to outer space.

## Supplementary information

ESM 1 (PDF 684 kb)

## Data Availability

Supplementary information includes three tables and accompanies this manuscript attached as a single file. RNA sequencing datasets are accessible through Gene Expression Omnibus under the accession number GSE239559. https://www.ncbi.nlm.nih.gov/geo/query/acc.cgi?acc=GSE239559.
