# Supplementary material for: Blood transcriptomics mirror regulatory mechanisms during hibernation—a comparative analysis of the Djungarian hamster with other mammalian species
Source: Pflugers Arch. 2023 Aug 5;475(10):1149–60. doi: 10.1007/s00424-023-02842-8 (PMC10499953; doi:10.1007/s00424-023-02842-8)
Supplement: Supplementary file 1 — (PDF 684 kb) [file 424_2023_2842_MOESM1_ESM.pdf]

Supplementary information

Blood transcriptomics mirror regulatory mechanisms during hibernation
- A comparative cross-species analysis

Pflügers Archiv – European Journal of Physiology

Valeria Rojas Cuyutupa, Dominique Moser, Victoria Diedrich, Yiming Cheng, Jean-Noël Billaud, Elena Haugg, Dominique Singer, Jürgen Bereiter-Hahn, Annika Herwig, Alexander Choukér

Corresponding authors:

Prof. Dr. Alexander Choukér
Laboratory of Translational Research 'Stress and Immunity'
Department of Anesthesiology
Ludwig Maximilians University Hospital
Marchioninistr. 15, 81377 Munich, Germany
Phone: +49.89.4400.76422; Fax: +49.89.4400.78886
E-mail: achouker@lmu.de

Prof. Dr. Annika Herwig
Institute of Neurobiology
Ulm University
Albert-Einstein-Allee 11, 89081 Ulm, Germany
Phone: +49.731.50.22630; Fax: +49.731.50.22629
E-mail: annika.herwig@uni-ulm.de

Suppl. table 1 Publications included for comparative literature analysis with TM_{IPA} derived from blood of Djungarian hamster at nadir of daily torpor (ZT04)

No.	Title of publication	Authors and Year	PMID	DOI
1	A functional transcriptomic analysis in the relict marsupial Dromiciops gliroides reveals adaptive regulation of protective functions during hibernation	Nespolo et al., 2018	30240506	DOI: 10.1111/mec.14876
2	Comparative tissue transcriptomics highlights dynamic differences among tissues but conserved metabolic transcript prioritization in preparation for arousal from torpor	Bogren et al., 2017	28332019	DOI: 10.1007/s00360-017-1073-x
3	Effect of torpor on host transcriptomic responses to a fungal pathogen in hibernating bats	Field et al., 2018	30080945	DOI: 10.1111/mec.14827
4	Effects of hibernation on bone marrow transcriptome in thirteen-lined ground squirrels	Cooper et al., 2016	27207617	DOI: 10.1152/physiolgenomics.00120.2015
5	Elevated expression of protein biosynthesis genes in liver and muscle of hibernating black bears (*Ursus americanus*)	Fedorov et al., 2009	19240299	DOI: 10.1152/physiolgenomics.90398.2008
6	Enhanced stability and polyadenylation of select mRNAs support rapid thermogenesis in the brown fat of a hibernator	Grabek et al., 2015	25626169	DOI: 10.7554/eLife.04517
7	Gene Expression Profiling in the Hibernating Primate, *Cheirogaleus Medius*	Faherty et al., 2016	27412611	DOI: 10.1093/gbe/evw163
8	Gut transcriptomic changes during hibernation in the greater horseshoe bat (*Rhinolophus ferrumequinum*)	Sun et al., 2020	32690984	DOI: 10.1186/s12983-020-00366-w
9	Hepatic gene expression profiling of 5'-AMP-induced hypometabolism in mice	Zhao et al., 2011	21224422	DOI: 10.1152/physiolgenomics.00174.2010
10	Modulation of gene expression in hibernating arctic ground squirrels	Yan et al., 2007	17925484	DOI: 10.1152/physiolgenomics.00075.2007
11	Modulation of Gene Expression in Key Survival Pathways During Daily Torpor in the Gray Mouse Lemur, *Microcebus murinus*	Biggar et al., 2015	26093281	DOI: 10.1016/j.gpb.2015.03.001
12	Seasonal and Regional Differences in Gene Expression in the Brain of a Hibernating Mammal	Schwartz et al., 2013	23526982	DOI: 10.1371/journal.pone.0058427
13	Transcriptional changes in muscle of hibernating arctic ground squirrels (*Urocitellus parryii*): implications for attenuation of disuse muscle atrophy	Goropashnaya et al., 2020	32488149	DOI: 10.1038/s41598-020-66030-9

Suppl. table 2 Matching genes (148) of comparative literature analysis with TM_{IPA} for each species and organ (red arrow increased measurement; green arrow decreased measurement)

Species	Organ	Genes
**Thirteen lined squirrel**	bone marrow	ABL1↑, ACLY↓, ACP2↑, ADAR↑, AKT1↑, ALOX5↓, ATF5↑, ATG3↑, ATG9A↑, CD47↑, CMPK2↓, COMMD9↓, CXCR4↓, CXXC1↑, EHMT1↑, EWSR1↑, FCGR2B↓, FERMT3↑, FNTA↑, FURIN↑, GAB1↓, GNG5↑, GNL3↑, HMGB1↑, HSPA8↓, IFI16↓, ITGA2B↑, KRR1↓, MAP3K7↑, MASP2↑, MDM2↑, MED12↑, MEF2D↑, MSH2↑, NCAPH2↑, NR1D1↑, PARVG↓, PDCD10↑, PI4K2B↑, PLTP↑, PPP2CA↓, PRKCD↑, RBM15↓, SAMD9L↓, SF3B1↑, SHC1↓, SKI↑, SLC2A3↑, SMARCA5↓, SREBF2↑, SRSF2↓, STAT6↑, STIM1↑, TFRC↓, TIAL1↑, TLN1↑, TLR3↓, TLR4↓, TRAF6↑, TRIOBP↑, TXNIP↑, UBE2B↓, UBE2L6↓, UBP1↑, USP36↑, VEZT↓
	brown adipose tissue	ACLY↓, ADRB2↓, AGER↓, ALOX5↓, ANGPT2↓, ATF5↑, ATG3↑, ATG9A↑, AZI2↓, CCNG2↓, CD200↑, CDK2↑, CLIC5↓, COMMD9↓, CREM↓, CTNNB1↓, CXXC1↑, E2F5↓, EHMT1↑, FCGR2B↓, FCGR2B↓, FGL2↓, FURIN↑, GLO1↓, GNG5↑, GPX7↑, GSTZ1↑, HSPA4↓, HSPA8↓, IFI27↑, IFNAR1↑, KRR1↓, LAMTOR2↓, LDB1↑, MAP2K1↓, MAP3K7↑, MAP3K7↑, MASP2↑, MDH1↓, MED12↑, MEF2D↑, NCAPH2↑, NEDD8↓, NFKBIA↑, NR1D1↑, NR1D1↑, PARP9↓, PCNA↓, PDCD10↑, PDE4DIP↓, PIM1↑, PPARGC1B↑, PPM1K↓, PRKCD↑, PSMB9↓, RAB10↓, RBM15↓, RTP4↓, SBNO2↑, SERPINB9↓, SF3B1↑, SKI↑, SLC25A19↓, SMAD4↑, SRSF3↓, STIM1↑, TFRC↓, TIAL1↑, TIMP3↓, TLR3↓, TLR4↓, TNFSF10↓, TRAF6↑, TXNIP↑, UBE2B↓, UBP1↑, USP36↑, VEZT↓, WDR77↓
	brain	BHLHE40↑, CCNG2↓, IFNAR1↑, ISG15↓, KIF3B↓, PNO1↓, SBNO2↑, SRSF2↓, TLN1↑, TXNIP↑, TYK2↑, VEZT↓
	heart	CREM↓, CTNNB1↓, HSPA4↓, HSPA4↓, ITGA2B↑, LDB1↑, MDH1↓, NEDD8↓, NOD2↓, PDCD10↑, PDE3A↑, PDE3A↑, PPARGC1B↑, PPM1K↓, RBM15↓, SMARCA5↓, SRSF2↓, TFAP4↑, TIMP3↓, TLR3↓, TLR4↓, TRAF6↑, TXNIP↑, TYK2↑
	liver	ADRB2↓, BCL2L13↑, CMPK2↓, CTNNB1↓, HSPA4↓, HSPA8↓, MASP2↑, NFKBIA↑, PIM1↑, PNO1↓, TIMP3↓, TRAF6↑, TXNIP↑, UBE2B↓

	skeletal muscle	AGER↓, AKT1↑, ATF5↑, CCL27↓, CREM↓, CTNNB1↓, DGKD↑, FGL2↓, GLO1↓, HSPA4↓, HSPA8↓, ITGA2B↑, KRR1↓, LDB1↑, MASP2↑, MDH1↓, PDE4B↑, PNO1↓, PPARGC1B↑, PPP2CA↓, PRKACA↓, PSMB9↓, RBM15↓, SF3B1↑, SKI↑, SLC25A19↓, SMARCA5↓, SPHK1↓, TFAP4↑, TFRC↓, TIAL1↑, TIMP3↓, TLR3↓, TLR4↓, TRAF3↑, UBE2B↓, VEZT↓
**Arctic ground squirrel**	brown adipose tissue	HMGB1↑, TIAL1↑
	liver	CPB2↑, HMGB1↑, TIAL1↑
	skeletal muscle	CREM↓, CXCR4↓, NR1D1↑, PPARGC1B↑, PPM1K↓, PPP2CA↓, TIAL1↑
**Mouse**	liver	BHLHE40↑, FOS↓, IFNB1↓, IL6↓, PAX5↑, PDE4B↑, PSMB9↓, TXNIP↑
**Gray Mouse Lemur**	heart	HSPA8↓
	kidney	HSPA8↓
	skeletal muscle	HSPA8↓
**Dwarf lemur**	white adipose tissue	GNL3↑, STIM1↑
**Little brown myotis**	wing tissue	ACP2↑, ADAM19↑, ADAR↑, AHR↓, AIP↑, AZI2↓, BCL2L11↓, BCL2L13↑, CD200↑, CD47↑, CD69↓, CD83↓, COMMD9↓, CXXC1↑, DGKD↑, E2F5↓, EHMT1↑, EWSR1↑, F2R↓, FERMT3↑, FGL2↓, GLO1↓, GPX7↑, GSTZ1↑, HSP90AA1↓, HSPA4↓, IFNAR1↑, KIF3B↓, LAMTOR2↓, LDB1↑, LTA↑, MAP2K1↓, MDH1↓, MED12↑, MEF2D↑, NFKBIA↑, NR1D1↑, PCNA↓, PDE3A↑, PDE4B↑, PHB↓, PIM1↑, PPM1K↓, PPP2CA↓, PRF1↓, PRKCD↑, RAB10↓, RELB↑, SBNO2↑, SLC17A7↑, SMARCA5↓, SMC2↓, SORT1↑, SPHK1↓, SREBF2↑, STAT6↑, STIM1↑, TIRAP↓, TLN1↑, TRAF1↑, TRIM25↑, TRIOBP↑, TYK2↑, USP36↑, VEZT↓

**Greater horseshoe bat**	gut	ACLY↓, ADRB2↓, CCNG2↓, CXCR4↓, CXCR6↓, FOS↓, GSTZ1↑, GZMB↓, ISG15↓, LY86↓, NR1D1↑, PARVG↓, PRF1↓, SRSF2↓, TXNIP↑, UBE2L6↓
**Black bear**	liver	EWSR1↑, HSPA8↓, TRIM25↑
	skeletal muscle	TXNIP↑
**Monito del monte**	brain	CCNG2↓, CXCR4↓, GNL3↑, TXNIP↑
	liver	DGKD↑, HSPA4↓, SPHK1↓, SRSF3↓, TXNIP↑
	skeletal muscle	TXNIP↑

Suppl. table 3 Assignment of 148 matching TM_{IPA} to physiological categories and processes (red arrow increased measurement; green arrow decreased measurement)

Category	Function/Disease	Genes
**Basic cellular mechanisms**	Transcription	ABL1↑, ADAM19↑, ADRB2↓, AGER↓, AHR↓, AKT1↑, ATF5↑, AZI2↓, BHLHE40↑, CDK2↑, CREM↓, CTNNB1↓, CXCR4↓, CXXC1↑, E2F5↓, EHMT1↑, EWSR1↑, F2R↓, FCGR2B↓, FOS↓, GLO1↓, HMGB1↑, HSPA4↓, HSPA8↓, IFI16↓, IFI27↑, IFNAR1↑, IFNB1↓, IL6↓, LDB1↑, MAP2K1↓, MAP3K7↑, MDM2↑, MED12↑, MEF2D↑, NEDD8↓, NFKBIA↑, NOD2↓, NR1D1↑, PARP9↓, PAX5↑, PCNA↓, PDCD10↑, PIM1↑, PPARGC1B↑, PPP2CA↓, PRKACA↓, PRKCD↑, RBM15↓, RELB↑, SBNO2↑, SHC1↓, SKI↑, SMAD4↑, SMARCA5↓, SORT1↑, SREBF2↑, STAT6↑, TFAP4↑, TFRC↓, TIAL1↑, TIMP3↓, TLR3↓, TLR4↓, TNFSF10↓, TRAF1↑, TRAF3↑, TRAF6↑, TXNIP↑, UBE2B↓, UBP1↑, WDR77↓
	Metabolism of protein	ADAM19↑, AHR↓, AKT1↑, ALOX5↓, ANGPT2↓, ATF5↑, CPB2↑, CTNNB1↓, FCGR2B↓, FOS↓, FURIN↑, GZMB↓, HSP90AA1↓, HSPA8↓ IFNAR1↑, IFNB1↓, IL6↓, ISG15↓, KRR1↓, MAP2K1↓, MAP3K7↑, MDM2↑, NEDD8↓, PDCD10↑, PIM1↑, PLTP↑, PPARGC1B↑, PPP2CA↓, SAMD9L↓, SKI↑, SMAD4↑, SPHK1↓, SRSF3↓, STAT6↑, TIMP3↓, TLR4↓, TNFSF10↓, TRAF3↑, TRIM25↑, TYK2↑, UBE2B↓, USP36↑
	Cellular homeostasis	ABL1↑, ACLY↓, ACP2↑, ADRB2↓ AGER↓, AHR↓, AKT1↑, ALOX5↓, ANGPT2↓, ATF5↑, ATG3↑, ATG9A↑, BCL2L11↓, BCL2L13↑, BHLHE40↑, CD47↑, CD69↓, CD83↓, CDK2↑, CLIC5↓, CTNNB1↓, CXCR4↓, F2R↓, FCGR2B↓, FGL2↓, FOS↓, FURIN↑, GAB1↓, GZMB↓, HMGB1↑, HSP90AA1↓, HSPA4↓, HSPA8↓, IFNAR1↑, IFNB1↓, IL6↓, LTA↑, MAP2K1↓, MAP3K7↑, MDM2↑, MSH2↑, NCAPH2↑, NFKBIA↑, NOD2↓, NR1D1↑, PAX5↑, PDE3A↑, PDE4B↑, PIM1↑, PPP2CA↓, PRF1↓, PRKACA↓, PRKCD↑, PSMB9↓, RELB↑, SHC1↓, SKI↑, SMAD4↑, SMARCA5↓, SPHK1↓, SREBF2↑, STAT6↑, STIM1↑, TFAP4↑, TFRC↓, TIAL1↑, TIMP3↓, TLR3↓, TLR4↓, TNFSF10↓, TRAF3↑, TRAF6↑, TXNIP↑, TYK2↑, USP36↑
	Autophagy	ABL1↑, ACLY↓, ACP2↑, ADRB2↓, AGER↓, AKT1↑, ATF5↑, ATG3↑, ATG9A↑, BCL2L11↓, BCL2L13↑, CTNNB1↓, CXCR4↓, GAB1↓, HMGB1↑, HSP90AA1↓, HSPA8↓, IL6↓, MAP2K1↓, MAP3K7↑, MDM2↑, MSH2↑, NOD2↓, NR1D1↑, PRKACA↓, PRKCD↑, SHC1↓, SMAD4↑, SPHK1↓, SREBF2↑, STIM1↑, TIAL1↑, TLR3↓, TLR4↓, TNFSF10↓, USP36↑

	Apoptosis	ABL1↑, ACLY↓, ADAR↑, ADRB2↓, AGER↓, AHR↓, AIP↑, AKT1↑, ALOX5↓, ANGPT2↓, ATF5↑, ATG3↑, AZI2↓, BCL2L11↓, BCL2L13↑, BHLHE40↑, CCL27↓, CD200↑, CD47↑, CD69↓, CDK2↑, CREM↓, CTNNB1↓, CXCR4↓, DGKD↑, EHMT1↑, EWSR1↑, F2R↓, FCGR2B↓, FGL2↓, FNTA↑, FOS↓, FURIN↑, GAB1↓, GLO1↓, GNG5↑, GNL3↑, GPX7↑, GSTZ1↑, GZMB↓, HMGB1↑, HSP90AA1↓, HSPA4↓, HSPA8↓, IFI16↓, IFI27↑, IFNAR1↑, IFNB1↓, IL6↓, ISG15↓, LAMTOR2↓, LTA↑, MAP2K1↓, MAP3K7↑, MDH1↓, MDM2↑, MED12↑, MEF2D↑, MSH2↑, NCAPH2↑, NFKBIA↑, NOD2↓, NR1D1↑, PAX5↑, PCNA↓, PDCD10↑, PDE3A↑, PDE4B↑, PIM1↑, PPARGC1B↑, PPP2CA↓, PRF1↓, PRKACA↓, PRKCD↑, RELB↑, SERPINB9↓, SF3B1↑, SHC1↓, SKI↑, SLC2A3↑, SMAD4↑, SMARCA5↓, SORT1↑, SPHK1↓, SREBF2↑, SRSF2↓, STAT6↑, STIM1↑, TFAP4↑, TFRC↓, TIAL1↑, TIMP3↓, TIRAP↓, TLN1↑, TLR3↓, TLR4↓, TNFSF10↓, TRAF1↑, TRAF3↑, TRAF6↑, TRIM25↑, TXNIP↑, UBE2B↓, USP36↑
	Necrosis	ABL1↑, ACLY↓, ADAR↑, ADRB2↓, AGER↓, AHR↓, AIP↑, AKT1↑, ALOX5↓, ANGPT2↓, ATF5↑, ATG3↑, BCL2L11↑, BCL2L13↑, BHLHE40↑, CCL27↓, CD200↑, CD47↑, CD69↓, CDK2↑, CPB2↑, CREM↓, CTNNB1↓, CXCR4↓, DGKD↑ EHMT1↑, EWSR1↑, F2R↓, FCGR2B↓, FGL2↓, FNTA↑, FOS↓, FURIN↑, GAB1↓, GLO1↓, GNL3↑, GPX7↑, GSTZ1↑ GZMB↓, HMGB1↑, HSP90AA1↓, HSPA4↓, HSPA8↓, IFI16↓, IFNAR1↑, IFNB1↓, IL6↓, ISG15↓, LAMTOR2↓, LTA↑ MAP2K1↓, MAP3K7↑, MDH1↓, MDM2↑, MED12↑, MEF2D↑, MSH2↑, NFKBIA↑, NR1D1↑, PAX5↑, PCNA↓, PDE3A↑, PDE4B↑, PIM1↑, PNO1↓, PPARGC1B↑, PPP2CA↓, PRF1↓, PRKACA↓, PRKCD↑, RELB↑, SERPINB9↓, SF3B1↑, SHC1↓, SKI↑, SLC25A19↓, SLC2A3↑, SMAD4↑, SMARCA5↓, SORT1↑, SPHK1↓, SREBF2↓, SRSF2↓, STAT6↑, STIM1↑, TFAP4↑, TFRC↓, TIAL1↑, TIMP3↓, TIRAP↓, TLN1↑, TLR3↓, TLR4↓, TNFSF10↓, TRAF1↑, TRAF3↑, TRAF6↑, TRIM25↑, TXNIP↑, TYK2↑, USP36↑

**Immune system**	Lymphopoiesis	ABL1↑, AHR↓, AKT1↑, BCL2L11↓, BHLHE40↑, CD69↓, CD83↓, CDK2↑, CTNNB1↓, CXCR4↓, FCGR2B↓, FGL2↓, FOS↓, FURIN↑, GZMB↓, HMGB1↑, HSP90AA1↓, IFNAR1↑, IFNB1↓, IL6↓, LTA↑, MAP2K1↓, MAP3K7↑, MDM2↑, MEF2D↑, MSH2↑, NCAPH2↑, NFKBIA↑, PAX5↑, PIM1↑, PRF1↓, PRKCD↑, PSMB9↓, RBM15↓, RELB↑, SHC1↓, SKI↑, SMARCA5↓, STAT6↑, STIM1↑, TFAP4↑, TLN1↑, TLR3↓, TLR4↓, TNFSF10↓, TRAF3↑, TRAF6↑, TYK2↑
	Recruitment of leukocytes	ADRB2↓, AGER↓, AHR↓, ALOX5↓, ANGPT2↓, BHLHE40↑, CCL27↓, CD200↑, CD47↑, CD69↓, CPB2↑, CTNNB1↓, CXCR4↓, FCGR2B↓, FERMT3↑, FOS↓, HMGB1↑, IFNAR1↑, IL6↓, LAMTOR2↓, LTA↑, NFKBIA↑, NOD2↓, PDE4B↑ STAT6↑, TIRAP↓, TLR3↓, TLR4↓
	Migration of cells	ABL1↑, ADAM19↑, ADRB2↓, AGER↓, AHR↓, AKT1↑, ALOX5↓, ANGPT2↓, ATG3↑, ATG9A↑, BHLHE40↑, CCL27↓, CD200↑, CD47↑, CD69↓, CPB2↑, CTNNB1↓, CXCR4↓, DGKD↑, E2F5↓, EHMT1↑, F2R↓, FCGR2B↓, FERMT3↑ FGL2↓, FOS↓, FURIN↑, GAB1↓, GZMB↓, HMGB1↑, HSP90AA1↓, HSPA8↓, IFI16↓, IFNAR1↑, IFNB1↓, IL6↓, ISG15↓, ITGA2B↑, LAMTOR2↓, LDB1↑, LTA↑, MAP2K1↓, MAP3K7↑, MASP2↑, MDM2↑, MEF2D↑, NFKBIA↑, NOD2↓, NR1D1↑, PARP9↓, PAX5↑, PDCD10↑, PDE4B↑, PDE4DIP↓, PIM1↑, PLTP↑, PRF1↓, PRKACA↓, PRKCD↑ RELB↑, SHC1↓, SMAD4↑, SORT1↑, SPHK1↓, SREBF2↑, STAT6↑, STIM1↑, TFAP4↑, TIMP3↓, TIRAP↓, TLN1↑, TLR3↓, TLR4↓, TNFSF10↓, TRAF6↑, TYK2↑
	Quantity of T lymphocytes	ABL1↑, ADAR↑, AHR↓, ANGPT2↓, BCL2L11↓, BHLHE40↑, CD47↑, CD69↓, CD83↓, CTNNB1↓, CXCR4↓, FCGR2B↓, FOS↓, IFNAR1↑, IFNB1↓, IL6↓, LTA↑, MAP2K1↓, NFKBIA↑, NOD2↓, PIM1↑, PRF1↓, PSMB9↓, RELB↑, SHC1↓, SPHK1↓, STAT6↑, TLN1↑, TLR3↓, TLR4↓, TRAF3↑, TRAF6↑, TXNIP↑, TYK2↑
	Quantity of B lymphocytes	ABL1↑, AHR↓, AKT1↑, ANGPT2↓, BCL2L11↓, CD69↓, CXCR4↓, FCGR2B↓, FOS↓, IFNAR1↑, IL6↓, LTA↑, MDM2↑, MSH2↑, PAX5↑, PIM1↑, PRF1↓
	Activation of T lymphocytes	ABL1↑, AGER↓, AKT1↑, AZI2↓, BCL2L11↓, BHLHE40↑, CD47↑, CD83↓, CTNNB1↓, FCGR2B↓, FOS↓, HMGB1↑, HSPA4↓, IFNAR1↑, IFNB1↓, IL6↓, MAP3K7↑, NFKBIA↑, PRF1↓, RELB↑, SERPINB9↓, SMAD4↑, STAT6↑, TIRAP↓, TLN1↑, TLR3↓, TLR4↓, TNFSF10↓, TRAF1↑, TRAF3↑, TYK2↑

	Viral Infection	ABL1↑, ADAR↑, ADRB2↓, AGER↓, AHR↓, AIP↑, AKT1↑, ALOX5↓, ATF5↑, BCL2L11↓, CD200↑, CD69↓, CDK2↑, CLIC5↓, CTNNB1↓, CXCR4↓, CXCR6↓, EHMT1↑, F2R↓, FCGR2B↓, FERMT3↑, FGL2↓, FNTA↑, FOS↓, FURIN↑, GAB1↓, GZMB↓, HMGB1↑, HSP90AA1↓, HSPA4↓, HSPA8↓, IFI27↑, IFNAR1↑, IFNB1↓, IL6↓, ISG15↓, ITGA2B↑ MAP2K1↓, MAP3K7↑, MDH1↓, MDM2↑, MED12↑, MSH2↑, NEDD8↓, NFKBIA↑, NOD2↓, PARP9↓, PDE3A↑, PDE4B↑, PIM1↑, PPM1K↓, PRF1↓, PRKACA↓, PRKCD↑, PSMB9↓, RELB↑, SAMD9L↓, SERPINB9↓, SF3B1↑, SLC2A3↑, SPHK1↓, SREBF2↑, SRSF2↓, STAT6↑, TFAP4↑, TFRC↓, TIAL1↑, TLR3↓, TLR4↓, TNFSF10↓, TRAF3↑, TRAF6↑, TRIM25↑, TXNIP↑, TYK2↑, UBE2B↓, UBE2L6↓
**Other organs**	***Proliferation***	
	Angiogenesis	ADRB2↓, AGER↓, AHR↓, AKT1↑, ALOX5↓, ANGPT2↓, CDK2↑, CTNNB1↓, CXCR4↓, F2R↓, FERMT3↑, FOS↓, FURIN↑, GAB1↓, HMGB1↑, HSP90AA1↓, IFI16↓, IFNAR1↑, IL6↓, ISG15↓, LTA↑, MAP2K1↓, MAP3K7↑, MDM2↑, NFKBIA↑, PDCD10↑, PDE3A↑, PIM1↑, PPARGC1B↑, PRKCD↑, RAB10↓, RBM15↓, SHC1↓, SMAD4↑, SPHK1↓, STIM1↑, TIMP3↓, TLR3↓, TLR4↓, TNFSF10↓, TRAF6↑, UBP1↑
	Proliferation of heart cells	AKT1↑, CDK2↑, CTNNB1↓, GNL3↑, IL6↓, MAP2K1↓
	Proliferation of hepatocytes	AHR↓, AKT1↑, CDK2↑, CPB2↑, CTNNB1↓, CXCR4↓, FOS↓, IL6↓, LTA↑, NFKBIA↑, TLR3↓
	Proliferation of kidney cells	AGER↓, ANGPT2↓, CDK2↑, IL6↓, SHC1↓, TLR4↓
	Inflammation	
	Inflammation of heart	AGER↓, AHR↓, HMGB1↑, RELB↑, STAT6↑, TLR3↓, TRAF3↑
	Inflammation of liver	FCGR2B↓, FNTA↑, GSTZ1↑, HMGB1↑, IFNAR1↑, IFNB1↓, IL6↓, ISG15↓, LTA↑, NOD2↓, PDE3A↑, PDE4B↑, PRF1↓, RELB↑, SRSF2↓, STAT6↑, TFRC↓, TIAL1↑, TIMP3↓, TLR3↓, TLR4↓, TNFSF10↓, TRAF3↑

Nephritis	AGER↓, ALOX5↓, ANGPT2↓, BCL2L11↓, CDK2↑, FCGR2B↓, GPX7↑, IFNAR1↑, IL6↓, LTA↑, MDM2↑, NFKBIA↑, PDE3A↑, PDE4B↑, PIM1↑, PPP2CA↓, PRKCD↑, RELB↑, SBNO2↑, SMAD4↑, SPHK1↓, TLR3↓, TRAF3↑
Protective effects	
Apoptosis of cardiomyocytes	ADRB2↓, AKT1↑, BCL2L11↓, CDK2↑, CREM↓, IL6↓, MAP2K1↓, MAP3K7↑, MDM2↑, MEF2D↑, PIM1↑, PRKCD↑, SPHK1↓, TLR4↓, TXNIP↑
Infarction of heart	ADRB2↓, AGER↓, CD47↑, CXCR4↓, F2R↓, FCGR2B↓, GLO1↓, IL6↓, ITGA2B↑, LTA↑, MASP2↑, PDE3A↑, PIM1↑, TLR4↓
Liver lesion	ABL1↑, ACLY↓, ADAM19↑, ADAR↑, ADRB2↓, AGER↓, AHR↓, AKT1↑, ALOX5↓, ANGPT2↓, ATF5↑, ATG9A↑, BCL2L11↓, BHLHE40↑, CD200↑, CD47↑, CDK2↑, CLIC5↓, CPB2↑, CTNNB1↓, CXCR4↓, E2F5↓, EWSR1↑, F2R↓, FCGR2B↓, FERMT3↑, FGL2↓, FNTA↑, FOS↓, FURIN↑, GNL3↑, GSTZ1↑, GZMB↓, HMGB1↑, HSP90AA1↓, HSPA4↓, HSPA8↓, IFI16↓, IFI27↑, IFNAR1↑, IFNB1↓, IL6↓, ISG15↓, ITGA2B↑, KRR1↓, LAMTOR2↓, LTA↑, MAP2K1↓, MAP3K7↑, MASP2↑, MDH1↓, MDM2↑, MED12↑, MEF2D↑, MSH2↑, NFKBIA↑, NOD2↓, NR1D1↑, PARVG↓, PCNA↓, PDCD10↑, PDE3A↑, PDE4B↑, PDE4DIP↓, PI4K2B↑, PPARGC1B↑, PPP2CA↓, PRF1↓, PRKACA↓, PSMB9↓, RAB10↓, RELB↑, SAMD9L↓, SBNO2↑, SERPINB9↓, SF3B1↑, SHC1↓, SLC25A19↓, SLC2A3↑, SMAD4↑, SMARCA5↓, SMC2↓, SORT1↑, SPHK1↓, SREBF2↑, SRSF2↓, SRSF3↓, STAT6↑, STIM1↑, TFRC↓, TIAL1↑, TIMP3↓, TLR3↓, TLR4↓, TNFSF10↓, TRAF3↑, TRAF6↑, TRIM25↑, TRIOBP↑, TXNIP↑, TYK2↑, USP36↑
Liver Regeneration	AHR↓, CDK2↑, CREM↓, CTNNB1↓, IL6↓
Damage of kidney	ABL1↑, ADRB2↓, ALOX5↓, ANGPT2↓, CTNNB1↓, GSTZ1↑, IL6↓, NOD2↓, PDE3A↑, PDE4B↑, SMAD4↑, TLN1↑, TLR3↓, TLR4↓, TXNIP↑
